# The MR quality landscape in Europe

**DOI:** 10.1186/s13244-026-02280-x

**Published:** 2026-05-07

**Authors:** Simone Busoni, Anna Pichiecchio, Lara Cristiano, Andrew England, Edwin H. G. Oei, Peter Lundberg, Michelle C. Williams, Francesco Santini, Emanuele Neri, European Society of Radiology (ESR)

**Affiliations:** 1 Health Physics Department, AOU Careggi (Firenze University Hospital), Firenze, Italy; 2 Department of Brain and Behavioural Disorders, University of Pavia, Pavia, Italy; 3 IRCCS Mondino Foundation, Pavia, Italy; 4 Pediatric Neurology Unit, Fondazione Policlinico Universitario ‘‘A. Gemelli’’, IRCCS, Rome, Italy; 5 Discipline of Medical Imaging & Radiation Therapy, University College Cork, Cork, Ireland; 6 European Federation of Radiographer Societies (EFRS), Cumiera, Portugal; 7 Department of Radiology & Nuclear Medicine, Erasmus MC, University Medical Center, Rotterdam, The Netherlands; 8 Clinical Department of Medical Radiation Physics, Region Östergötland, Linköping, Sweden; 9 Clinical Department of Radiology in Linköping, Region Östergötland, Linköping, Sweden; 10 Center for Medical Image Science and Visualization (CMIV), Linköping University, Linköping, Sweden; 11 Department of Health, Medicine, and Caring Sciences, Linköping University, Linköping, Sweden; 12 British Heart Foundation Centre for Research Excellence, University of Edinburgh, Edinburgh, United Kingdom; 13 Basel Muscle MRI, Department of Biomedical Engineering, University of Basel, Basel, Switzerland; 14 Department of Radiology, University Hospital Basel, Basel, Switzerland; 15 Academic Radiology, Department of Translational Research, University of Pisa, Pisa, Italy; 16 European Society of Radiology, Am Gestade 1, Vienna, Austria

**Keywords:** Magnetic resonance imaging, Quality assurance, health care, Quality control, Education, Guideline adherence

## Abstract

**Objectives:**

Over the last few years, with the introduction of advanced MR imaging techniques, increasing exam demand and the growth of multi-center clinical trials and artificial intelligence (AI)-driven analysis, it has become increasingly difficult to guarantee image quality across time and institutions. Quality Assurance (QA) and Quality Control (QC) programs have therefore become essential. The aim of the survey was to map how MRI QA and QC are implemented in routine clinical practice and, where applicable, in research settings, across Europe, to identify the points where harmonization, coordination, or further education is needed.

**Materials and methods:**

An anonymous survey was distributed between October and December 2024 through ESR, EFOMP, EFRS member societies and ESMRMB to healthcare professionals, addressing five broad categories: characteristics of participants and their institutions, national MRI QA/QC guidelines/legislation and awareness, local organization for MRI QA, local (institute level) organization for MRI system performance QCs, conventional imaging QCs and qMRI QCs.

**Results:**

269 responses were obtained from 37 different countries. Respondents were radiologists (52%), followed by Medical Physics Experts/Physicists/Engineers (30%), and radiographers (17%). Only a few countries have mandated national legislation addressing MRI QA/QC, while many others rely on voluntary guidelines or lack formal protocols. Most respondents recognized the importance of robust QA/QC programs. There is a strong consensus among respondents on the need for harmonized guidelines from organizations like ESR, multidisciplinary collaboration, and easily accessible training.

**Conclusions:**

The European landscape regarding MRI quality is very heterogeneous, with different regulations across countries, and different penetration of MRI QA and QC training and regulation. The European Society of Radiology is optimally positioned with partners to play an active role in the harmonization of MRI quality education and practices across Europe, and we propose the development of a set of recommendations for MRI quality control and assurance.

**Critical relevance statement:**

There is scope for raising awareness of both MRI Quality Control (QC) and Quality Assurance (QA) issues and improvement in these fields to ensure patient safety, reduce diagnostic errors, and allow more patients to benefit from MR imaging.

**Key Points:**

- Our survey of MRI QA and QC practices across Europe revealed significant heterogeneity in regulations and practices between countries and institutions.
- There is a widespread lack of awareness and implementation of MRI quality guidelines.
- The ESR MR Safety and Quality Committee advocates for the standardization and enhancement of MRI quality training for all professionals involved in this issue.

**Graphical Abstract:**

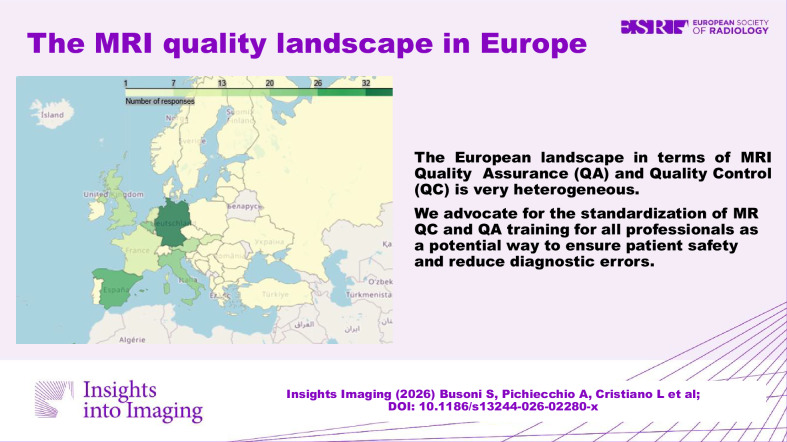

## Introduction

Magnetic resonance imaging (MR) is the method of choice for imaging the human body without using ionizing radiation and offers, at the same time, excellent soft-tissue contrast and high-resolution morphological, metabolic and functional images. Therefore, MR plays a crucial role in the diagnostic work-up of neurological, musculoskeletal, cardiovascular, and oncologic disorders.

Over the last few years, with the introduction of advanced imaging techniques (e.g., quantitative MR, functional MR), increasing exam demand and the growth of multi-center clinical trials and AI-driven analysis, it has become increasingly difficult to guarantee image quality across time and across institutions [1, 2]. Quality assurance (QA) and quality control (QC) programs have therefore become essential. QA in MR comprises all the management practices developed by the MR imaging team led by the MR supervising radiologist to ensure that every imaging procedure is necessary and appropriate to the clinical problem and that the images generated contain information critical to the solution of the problem. QC refers to a series of distinct and periodic technical quality control measurement procedures that ensure the production of a satisfactory product, in this case, high-quality diagnostic images, and is an integral part of quality assurance [3]. The lack of such measures can lead to poor image quality, increased need for repeat scans, diagnostic errors, and ultimately, substandard patient care [4].

International organizations such as the American College of Radiology (ACR) and the International Electrotechnical Commission (IEC) have published standards for MR QA and QC [3, 5, 6]. However, the European MR quality landscape is heterogeneous and, even for safety guidelines, different countries implement variations of the international safety guidelines, sometimes at a normative/legislative level, and sometimes as non-binding national recommendations [7]. Prior studies have noted substantial differences in how QA and QC are performed across institutions and countries, ranging from well-established national guidelines to a complete absence of formal protocols [8].

In recent years, the increasing use of quantitative MR (qMR) and AI-assisted image analysis has further highlighted the importance of regulating QA/QC procedures [9–14]. Accurate and reproducible quantitative data (such as relaxometry parameters, iron concentration, or proton density fat fraction) require rigorous monitoring of scanner performance and software/hardware updates over time. Furthermore, there is growing awareness of the need for objective metrics in image quality assessment, moving beyond visual inspection alone [15].

To better understand the current state of QA and QC in routine clinical practice and, where applicable, in research settings, across European institutions, we, as the ESR MR Safety and Quality Subcommittee, conducted a comprehensive survey among imaging professionals. This manuscript presents the results of that survey, offering a summary of current practices, perceived challenges, and potential directions for improvement in MR quality assurance and control across European institutions.

## Materials and methods

The survey was promoted by the ESR “MR Safety and Quality Subcommittee.” The 32 survey questions were developed through open discussion among members of the ESR ‘MR Safety and Quality Subcommittee.’ The selection process accounted for a potential heterogeneity across the European landscape, focusing on MR quality management as well as regulatory, operational, and clinical issues. To encourage participation, the survey was designed to be completed in less than 30 min. The primary objective was to provide the ESR with the necessary feedback to optimize future Education and Training (E&T) initiatives and to promote harmonization across ESR member states. An anonymous survey was developed and distributed via the SurveyMonkey platform (SurveyMonkey). The survey underwent pilot testing by the members of the ESR ‘MR Safety and Quality Subcommittee’ prior to distribution. This was undertaken to ensure technical functionality, confirm that the questions were unambiguous, and verify that the completion time was within the expected 30-min limit.

The survey questionnaire was distributed through ESR member societies to healthcare professionals and was available for 6 weeks from November 2024 to the end of December 2024.

The survey was distributed to the European Full and Allied Science Members in the ESR with the following professions: Medical Physics Expert, Medical Physicist, Radiographer and Radiologist. This totaled 27,475 members across 51 countries. Additionally, the survey was promoted through the ESR National Societies Committee Delegates, which included the presidents of all 47 National Institutional Members of the ESR, the Quality, Safety, and Standards Committee Delegates, encompassing National and Subspecialty Society Representatives, totaling 53 societies. Moreover, the survey was distributed via the EFRS, which represents approximately 100,000 radiographers across Europe, the EFOMP, which has 9100 declared members, and the ESMRMB, which has fewer than 1000 members. While overlap is possible between the members of the societies, we estimate that the potential reach of the survey was between 100,000 and 150,000 individuals in at least 51 countries. It is important to note that this does not constitute a representative sample of the population of radiology professionals in Europe in the statistical sense, as no sampling strategy aimed at preventing bias was implemented. In particular, only a subset of recipients has an active role in MR, and plausibly, only an answer from each institute was provided.

To better specify the target of the survey, the following definitions were to be considered by participants, as defined by the ACR:

- Quality assurance (QA): comprises all the management practices developed by the MR imaging team led by the MR supervising radiologist to ensure that every imaging procedure is necessary and appropriate to the clinical problem and that the images generated contain information critical to the solution of the problem.
- Quality control (QC): a series of distinct technical procedures that ensure the production of a satisfactory product, in this case, high-quality diagnostic images, and is an integral part of quality assurance.

The questions were divided into five broad categories:

1. Characteristics of participants and their institutions,
2. National MR QA/QC guidelines/legislation and awareness,
3. Local organization for MR QA,
4. Local (institute level) organization for MR system performance QCs, including both conventional QCs and qMR QCs,
5. Conventional imaging QCs and qMR QCs contents.

Feedback/Opinions on MR QA/QC topics were also requested from participants. While the survey was targeted at the countries affiliated with the European societies, no specific restriction was posed in the definition of “European.” However, the respondent indicated the country where their institution was located.

**Fig. 1:**
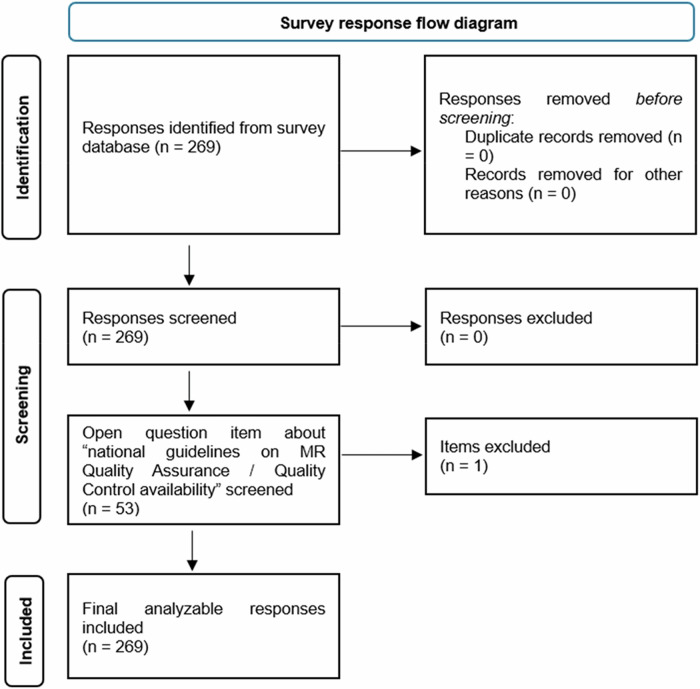
Survey response selection flow diagram

Data collected through the online survey platform were systematically organized into a dedicated database using Microsoft Excel (Microsoft Corp). Each survey response consisted of several fields, including both categorical variables and open-ended text items. The entries were exported into an Excel spreadsheet, where each column represented a specific variable, and each row represented a single respondent. Before the analysis, the data were screened to ensure integrity; specifically, responses were manually verified so that no duplicate entries were present. The survey response flow diagram is illustrated in Fig. 1. To ensure the interpretability of the responses to open-ended questions, a thematic analysis was performed, identifying recurring patterns and key concepts. Descriptive statistics are presented as absolute frequencies and percentages for categorical data. Results that were incomplete were only accepted if they contained more than the basic demographic information. Data analysis was performed using descriptive statistics with Microsoft Excel 365 (Microsoft).

## Results

### Demographics

**Fig. 2:**
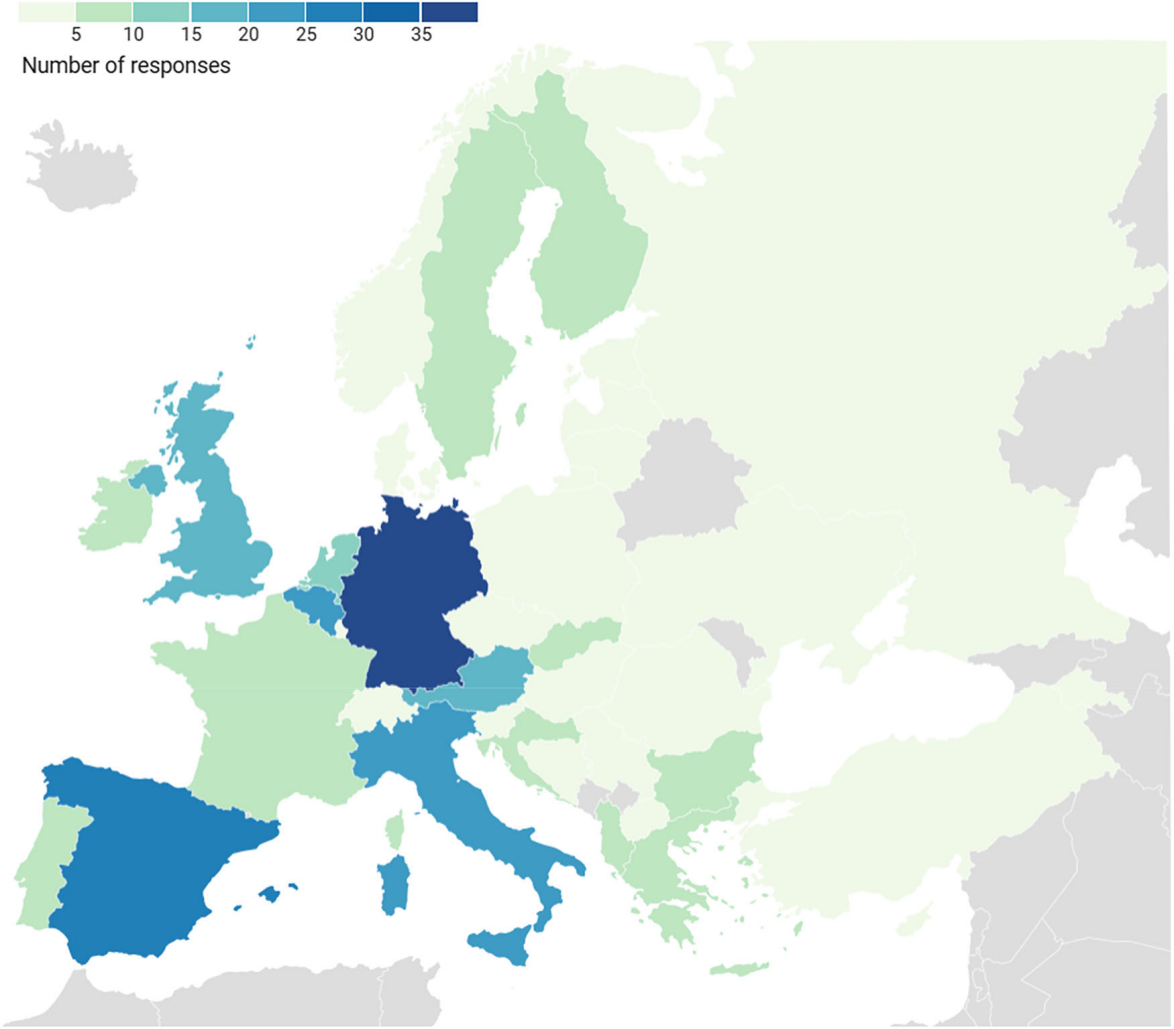
Number of valid responses received by country

269 responses were obtained from 37 different countries (Fig. 2), resulting in a representation of 72% of the countries directly contacted by the ESR. Details of respondents’ professions and their types of institution are presented in Table 1a, b.

**Table 1:** Percentage of respondents’ professions (**a**) and percentage of respondents from University/Research Hospitals or outpatient facilities (**b**)

a. Respondents’ profession	
Radiologist	53.5%
Medical physics expert/physicist/engineer	29.8%
Radiographer	16.7%

b. Respondents’ institution type	
University/research hospital	59.9%
Non-university/non-research hospital	32.3%
Outpatient facility	7.8%

The respondents’ institutions were mostly equipped with 1.5-T MR scanners (232 responses, 52.5% of the total cited) and/or 3-T scanners (167, 37.7%), with 27 respondents (6.1%) indicating possession of scanners with field strength below 1.5 T and 17 respondents (3.8%) indicating possession of scanners above 3 T.

### National legislation and QA program and awareness

Only 30% of respondents (78 individuals) indicated the existence of national MR QA/QC guidelines, and 19% (52) provided specific references that, after a thematic analysis and recurring patterns identification, were summarized into 29 specific national guidelines to be considered as respondents ‘assumptions. Only Italy provided evidence of a specific MR Quality Assurance/Quality Control concept/framework/program mandated by law [9]. 30% of the respondents were not aware at the time of the survey of the existence of national legislation.

As for the availability of national guidelines on MR quality assurance/quality control, the responses were positive, negative, and unaware in 30%, 48%, and 22% of cases, respectively.

A total of 29 references to specific national guidelines were provided, with only Italy and Germany referring to national institutional guidelines, while most of the other references pertained to scientific associations (see Table 2).

**Table 2:** Links to national QA and QC guidelines, as reported by the respondents

Country	Documents
Austria	mpMRI prostate: https://www.oerg.at/quality-assurance/
Belgium	BQuadril
France	Rapport SFPM n°23 : Contrôle de qualité specifique en IRM
France	MRI QC MANUAL (ACR)
Germany	“Quality requirements for MRI simulation in cranial stereotactic radiotherapy: a guideline from the German Taskforce ‘Imaging in Stereotactic Radiotherapy’”. 10.1007/s00066-023-02183-6
Germany	AAPM Task group 284 report: magnetic resonance imaging simulation in radiotherapy: considerations for clinical implementation, optimization, and quality assurance
Germany	“Guideline on criteria for quality assessment in magnetic resonance imaging according to Section 135b Paragraph 2 SGB V – QBK-RL”. https://www.g-ba.de/richtlinien/21/ https://www.g-ba.de/downloads/62-492-2036/QBK-RL_2019-10-17_iK_2020-01-01.pdf
Germany	Quality assessment guideline for magnetic resonance imaging https://www.g-ba.de/richtlinien/21/
Germany	GUFI Guidelines on Ultrahighfield MRI Systems
Germany	DIN EN IEC 62464-1:2018
Germany	“Leitlinien der Bundesärztekammer zur Qualitätssicherung der Magnet-Resonanz-Tomographie”
Germany	“Richtlinien der Kassenärztlichen Bundesvereinigung”
Germany	Bundesärztekammer
Greeece	ΕΣΥΔ ΚΟ-ΑΠΕΙΚ_ΕΡΓ 01/01/31-3-2022
Italy	Decreto Ministeriale 14 gennaio 2021: Determinazione degli standard di sicurezza e impiego per le apparecchiature a risonanza magnetica e individuazione di altre tipologie di apparecchiature a risonanza magnetica settoriali non soggette ad autorizzazione. Gazzetta Ufficiale Serie Generale n. 65 del 16-03-2021. https://www.gazzettaufficiale.it/eli/id/2021/03/16/21A01353/sg (Ministry of Health Decree on 14th January 2021)
Italy	AIFM (Italian Association of Medical and Health Physics) Report n.2 “Raccomandazioni per l’assicurazione di qualità in risonanza magnetica” https://www.fisicamedica.it/oldsite/sites/default/files/documenti/2004_n2_ReportAIFM.pdf
Italy	AIFM (Italian Association of Medical and Health Physics) Report n.12 “Assicurazione di qualità in DWI” https://www.fisicamedica.it/oldsite/aifm/documenti/report/2017/report-12-qa-dwi
Italy	Indicazioni operative INAIL - gestione sicurezza e qualità in Risonanza Magnetica https://www.inail.it/content/dam/inail-hub-site/documenti/2021/06/alg-attuazione-dei-nuovi-standard-sicurezza.pdf
Italy	SIRM (Italian Society of Medical and Interventional Radiology) MRI guideline https://sirm.org/wp-content/uploads/2025/06/Protocolli-di-Risonanza-Magnetica-per-indicazione-clinica.pdf https://sirm.org/wp-content/uploads/2021/04/325-Documento-SIRM-2021-DM-Salute-14-gennaio-2021-Sicuerazza-RM-Sinossi-per-il-Radiologo.pdf
Serbia	https://mos-medsestra.ru/biblioteka/actual_docs/МР%20Основы%20безопасности%20при%20проведении%20МРТ%20от%202019.pdf
Spain	Protocolo RM Seram
Spain	Protocolo Español de control de calidad en radiodiagnóstico 2011
Sweden	https://swedrad.se/mrsakerhet (Swedish Alliance for MR safety)
Sweden	QC: https://ledsys.lio.se/Document/Published/25368 (QC in MR)
The Netherlands	Leidraad Kwaliteitscontrole Radiologische Apparatuur - NVKF (https://nvkf.nl/sites/default/files/LeidraadKwaliteitscontroleRadiologischeApparatuur3.01.pdf)
The Netherlands	https://radiationdosimetry.org/ncs/mri-qa-for-rt
Türkiye	Tıbbi Cihazların Test Kontrol ve Kalibrasyonu Hakkında Yönetmelik
UK	IPEM Report 112: Quality Control and Artifacts in MRI
UK	MHRA
UK	https://www.gov.uk/government/publications/safety-guidelines-for-magnetic-resonance-imaging-equipment-in-clinical-use

The authors take no responsibility regarding whether these guidelines are officially endorsed by the respective professional societies. This list is based on self-reporting by the respondents of the survey and might not be complete

### Local quality assurance (QA) program implementation and responsibilities

A total of 48.5% of respondents reported having a QA program implemented at the institutional or local level. In contrast, 41.2% indicated that no QA program is in place, while 10.3% were unaware at the time of the survey of the existence of such a program. According to a multiple-choice question, those responsible for managing/leading the QA programs are listed in Table 3 with scanner manufacturer personnel representing 0.4%.

**Table 3:** Professionals leading imaging QA programs

Responsible for the quality assurance program	
Medical physics expert/physicist/engineer	69.3%
Radiologist	42.6%
Radiographer	32.6%

Multiple selections were allowed; therefore, percentages exceed 100%

QA actions are carried out: daily in 13.9% of cases, weekly in 11.8%, monthly in 23.8%, yearly in 45.5%, and less frequently than once per year in 5% of cases. The revision of QA procedures occurs more frequently than once per year in 18.8% of cases, every 1–2 years in 40.6%, every 2–5 years in 24.7% not performed or unknown in 15.8% of cases.

### Local implementation of the QC program

A total of 49% of respondents reported having an image quality control program implemented at the institutional or local level. In contrast, 41% indicated that no formal image quality control program is in place, while 10% were unaware of the existence of such a program. Conventional image quality protocols mainly address image homogeneity (83.6%), SNR performance (74.7%), coil functionality image homogeneity (66.4%), image ghost (55.5%), or slice thickness (55.5%).

**Fig. 3:**
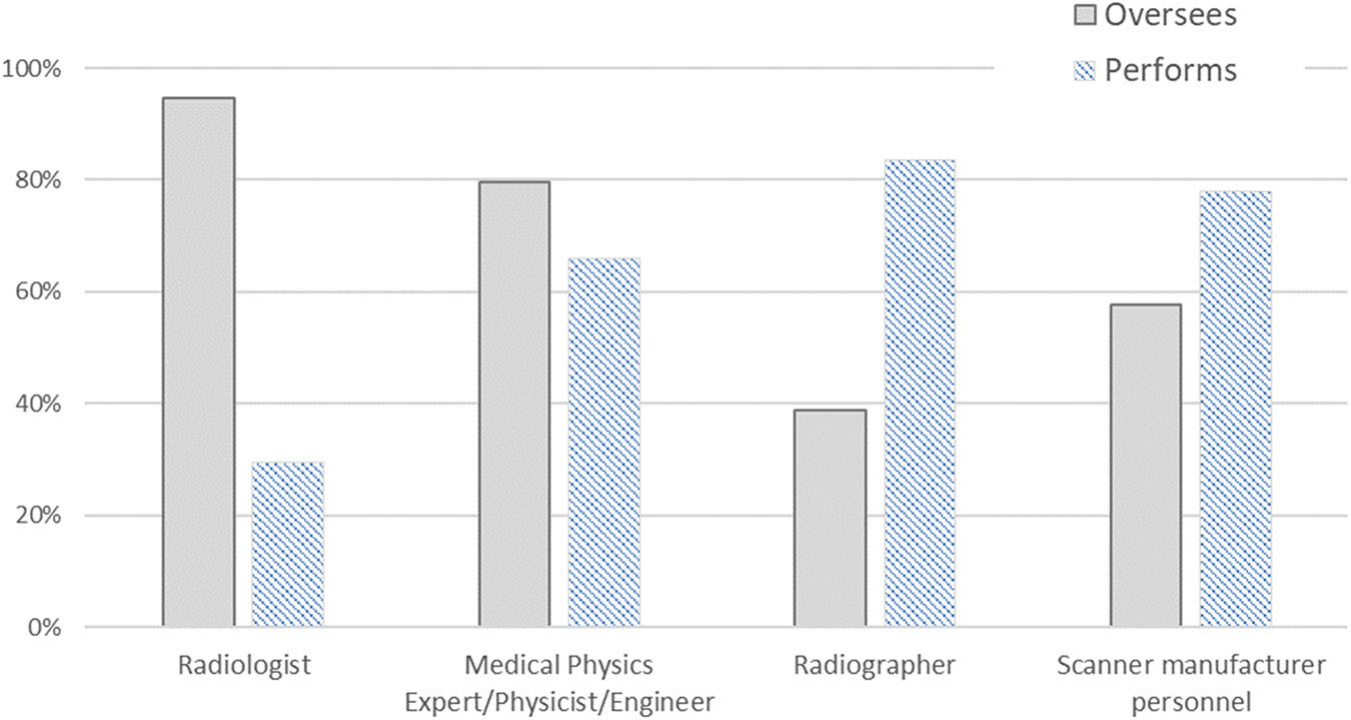
Professional roles responsible for overseeing and/or performing quality control procedures in MRI across survey participants

A multiple-selection question addressed the operational tasks of image quality QCs, investigating who oversees and performs QCs. Results are shown in the Fig. 3.

Image quality control is performed daily in 18.7% of cases, weekly in 17.3%, monthly in 22.7%, yearly in 40%, and less frequently than once per year in 1.3% of cases. The revision of QC protocol occurs more frequently than once per year in 19.4% of cases, every 1–2 years in 37.5%, every 2–5 years in 25.0% not performed or unknown in 18.0% of cases.

### Local implementation of QC program in qMR

**Fig. 4:**
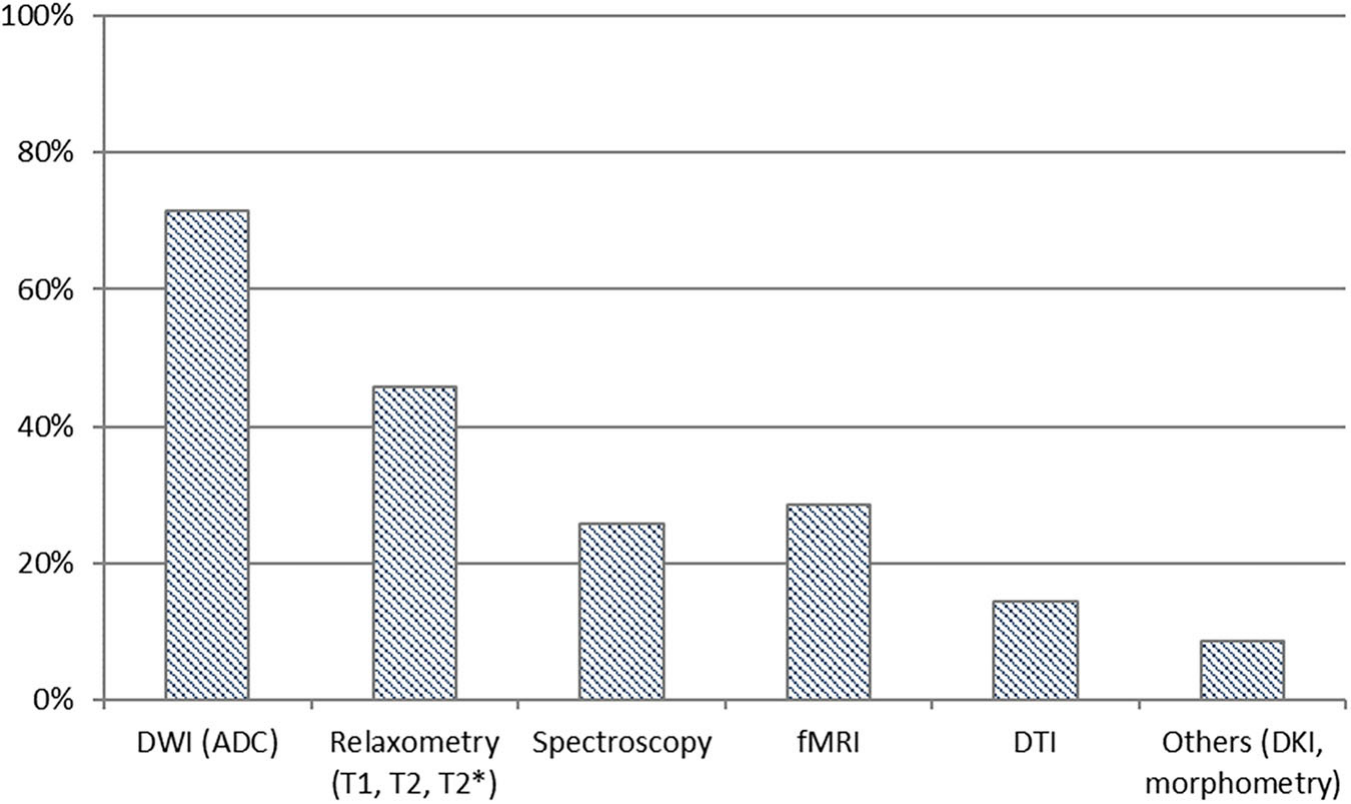
Distribution of quantitative MRI techniques and parameters covered by the local qMRI QC programs

A local, specific QC program devoted to quantitative MR (qMR) is established in only 22.2% of respondents’ institutions, while 77.8% do not have a qMR QC program. The qMR technique or parameter most commonly addressed by existing QC programs is listed in Fig. 4.

Regarding current practices, the use of test objects (phantoms) for quality control shows a notable lack of standardization. Most participants (81%) reported using manufacturer-provided phantoms, while only 3% reported the use of traceable phantoms specifically designed for quantitative MR (qMR). The most commonly used test objects include ACR phantoms (24%), custom-built phantoms (15.3%), Eurospin (3.6%), and NIST, Magphan, and FunStar phantoms (2.9% each). The Italian Association on Medical and Health Physics (AIFM) standard phantom is used by 2% of respondents, while QUASAR and fBIRN phantoms are used by 1.4% and 1%, respectively. This variability highlights a clear need for standardization to improve comparability in longitudinal and multi-center studies and to facilitate integration with advanced methodologies such as Artificial Intelligence (AI) and qMR.

### Multidisciplinary collaboration in QA aspects

A total of 148 participants (55% of survey respondents) answered a multiple-choice question regarding the availability of a multidisciplinary team to support the process of acquisition optimization and post-processing. Radiologists and radiographers commonly request support for exam optimization or the introduction of new sequences (63.5%), artifact management (60.8%), and post-processing in advanced imaging (50.7%), while 30.0% answered that there is no collaboration beyond safety aspects. A subset of these respondents (122 participants, 45% of survey respondents) specified the anatomical regions for which collaboration is most frequently requested, with the following distribution: brain (75.1%), abdomen (liver, pancreas, kidneys) (68.9%), pelvis (prostate and female pelvis) (65.6%), cardiovascular system (54.1%), musculoskeletal (MSK) (51.6%), and breast (45.9%). An additional 9% indicated collaboration for research, clinical trials, whole-body MR, stress/exercise CMR, fetal MR, or interventional MR.

A total of 56 radiologists (20% of survey respondents) reported that, in the clinical research field, they receive support from other professionals for post-processing analysis in 37% of cases. This support is provided mainly by medical physics experts (41%), physicists or engineers (34%), radiographers (12.5%), and others, such as manufacturer application specialists (12.5%). These professionals are based in radiology departments (54.7%), medical physics departments (28.3%), dedicated research units (9%), or manufacturer support structures (8%).

The collaboration occurs on a daily basis in 13% of cases, weekly in 16%, monthly in 20%, and occasionally in 51% of cases.

### Feedback from respondents

A total of 159 participants (58% of survey respondents) provided feedback concerning QA/QC practices and training. Among these, 94.3% expressed that a QA/QC program would be highly beneficial for daily clinical work, as it ensures the production of high-quality images, enhances patient safety, and supports the efficiency and accuracy of diagnostic processes.

The key advantages highlighted include:

- Consistent image quality
- Improved diagnostic accuracy
- Reduced errors and repeat scans
- Equipment performance monitoring
- Compliance with regulations
- Patient safety
- Optimized workflow

Overall, effective QA and QC programs are clearly viewed as highly essential for continuously improving the reliability, efficiency, and safety of MR imaging, thus enhancing both clinical outcomes and the overall patient experience.

Participants suggested that QA and QC programs should be implemented to assess and ensure system performance by: providing high diagnostic value and consistent image quality, standardizing performance across hospitals and examination times within hospital networks, enabling earlier detection of hardware/software issues, identifying subtle issues such as image/data quality drift, gaining control over post-processing procedures, measuring the impact of hardware/software updates or upgrades, monitoring system performance independently of manufacturer tests, supporting the installation and development of new methodologies, ensuring reproducible results and traceability in case of image/data abnormalities, improving the accuracy and reproducibility of quantitative MR data (e.g., T1, ADC, etc.)

Participants also expressed a strong desire for support and recommendations from the ESR and other relevant scientific associations. 92.7% of respondents believe comprehensive guidelines and standardized protocols would be beneficial, and 92.1% that ESR should provide specialized training courses on QA and Image Quality QC. These findings highlight the perceived importance of a multidisciplinary approach to QA programs and QC, emphasizing their crucial role in ensuring accurate diagnosis, optimizing protocols, enabling early fault detection, and enhancing patient safety.

## Discussion

This European survey sought to provide a structured overview of how MR Quality Assurance (QA) and Quality Control (QC) are currently addressed across different clinical and research settings. It highlights substantial heterogeneity in regulatory frameworks, institutional organization and professional awareness, showing that MR QA/QC practices remain patchy and inconsistent across the continent. In fact, although QA/QC is crucial for patient safety, accurate diagnoses and efficient imaging, only two out of 37 countries have regulations specifically addressing QA and QC in MR. Even fewer countries reported having guidelines tailored to quantitative MR (qMR) procedures.

Specifically, responders highlighted that only Italy, Bosnia, Germany, Greece, Portugal and Romania have legislation, but a specific legislation reference was provided only by Italian responders. The Italian current legislation, through the Ministerial Decree of January 14, 2021 [9], provides a regulatory framework that integrates safety with quality in the use of MR systems. It defines a quality assurance program for MR scanners, requiring periodic checks and documentation of performed verifications to ensure the diagnostic effectiveness of the equipment.

In Italy, two main professional figures are responsible for safety and quality control: a radiologist with proven experience in MR as Medical Director for Clinical Safety and Diagnostic Effectiveness and a professional with a degree in Physics or Engineering (a Medical Physicist in 90% of cases) and specific experience and training in the MR safety field who operates with role corresponding to MR Safety Expert (MRSE) and share with radiologist most of the tasks of MRSO (2017 INAIL data) [10].

The QA program includes periodic QC checks, ruled out at least every 6 months, and digital storage of the periodic check document. The two Italian safety and quality experts sign together acceptance tests and local safety rules, while the final responsibility of each medical exam lies with the radiologist on shift. This is not fully reflective of all practices across the European Union, and of all the actors involved in the QA and QC process (i.e., radiographers) and in other European legal systems, MR quality is not regulated by law and relies only on national guidelines. This lack of standardization could jeopardize data interoperability, data reproducibility in research fields, and cross-border patient care.

Strong and effective QA and QC processes directly improve MR image quality, reproducibility, and clinical reliability. Conversely, poor acquisition protocols, as well as systems with inferior technical performance, can lead to diagnostic mistakes, unnecessary scans, and negative patient outcomes [16, 17]. Authoritative guidelines and standards like those from the American College of Radiology (ACR) and International Electrotechnical Commission (IEC) stress that high technical standards are vital, particularly for longitudinal studies and for producing reliable quantitative results [3, 5]. Besides ensuring high diagnostic standards, QA and QC programs help catch subtle yet meaningful issues—such as hardware and coil failures or step changes associated with software updates—that can impact image parameters. This is especially true in busy imaging centers, where small deviations can affect hundreds of patient scans before they are detected and rectified [18].

Our survey highlights that most European countries lack clear national requirements for MR QA and QC. There is also a concerning lack of awareness: at the time of the survey, one-third of respondents did not know whether national legislation existed, and 22% were unaware of national guidelines.

Notably, the survey revealed that, within a single country, respondents often rely on different guidelines or protocols for MR QA and QC. Several participants noted that this variation can arise from the coexistence of institutional, regional, and association-based recommendations, sometimes resulting in differing approaches even among professionals working in similar settings. This variability further emphasizes the fragmented nature of MR QA and QC practices across Europe, even within individual countries. It also reinforces the need for harmonized, nationally-endorsed standards and comprehensive educational efforts to promote uniformity in quality assurance and control protocols.

A key issue is the insufficient application of QC protocols for quantitative MR. Techniques like T1 and T2 mapping, diffusion imaging, and MR spectroscopy are increasingly common, especially in clinical trials and neuroimaging, but they are particularly sensitive to equipment variability such as scanner drift, magnetic field variation, and software updates [5]. International standardization efforts, including ISMRM/NIST phantoms and fBIRN calibration, have shown that consistent qMR protocols and phantom-based validation boost reliability across sites [19, 20]. However, everyday uptake of these tools is slow, mainly due to the need for further training and limited support across vendors.

Automation is a promising solution to many practical barriers. Research shows that AI-based image assessment and automated phantom measurements reduce manual workload and increase reliability and sensitivity to changes [21, 22]. AI-based methods could potentially also detect declining scanner performance earlier, leading to preventative maintenance and less downtime. Still, introducing AI procedures and other automation requires investments in technology, new staff training, and preferably also collaboration with manufacturers. Currently, manual and semi-automated QC checks are still the norm, remaining vulnerable to inconsistencies and human error, although automation is possible [23].

Feedback from survey participants revealed a clear demand for coordinated action at a European level. Many agreed that the ESR and related professional organizations should offer unified QA and QC guidelines, develop training resources, and support certification for QA and QC professionals continent-wide. Campaigns like EuroSafe Imaging have already proven that collective resources and benchmarking can improve CT practices across the EU [24]. Building robust QA and QC in MR will require transparent collaboration among healthcare professionals (radiologists, medical physics experts/medical physicists, radiographers), IT experts, and industry partners, all of whom need to work together to deliver effective standards, training, and interoperable tools, including vendor-neutral/standardized testing phantoms and a solid metrological approach to qMR [25–27]. QA/QC programs should also address hybrid modalities (PET/MR) and emerging applications as LINAC/MR.

When interpreting these findings, a few limitations should be considered. First, participation in the survey was voluntary, raising the possibility of selection bias toward individuals and institutions already engaged in or interested in QA/QC. Second, the data are based on self-reported practices, which may not fully reflect what actually happens in practice. Finally, even with a broad geographic reach, some regions may still be underrepresented, which could influence the overall picture.

### Strategic directions

Beyond providing a snapshot of current practice, this survey aims to help identify priority actions for the European radiological community. The findings point toward several clear strategic directions.

1. First, there is a strong need for harmonized European guidance that defines a minimum set of MR QA and QC requirements applicable across vendors, field strengths, and clinical settings. This would not replace existing national regulations but would provide a common framework to support reducing variability and lead to more consistent image quality across Europe. Experience from other imaging modalities has shown that coordinated, society-led European initiatives can play an important role in improving quality and safety [24].
2. Second, education and training in both QA and QC also need to be strengthened. QA/QC should be recognized as a core competency for all professionals involved in MR. Structured, European-level training programs with clear competencies could improve awareness, support routine implementation and reduce disparities between institutions with different resources. Recent studies suggest that targeted training initiatives can meaningfully improve consistency and reliability, particularly in advanced MR applications [13].
3. Third, a more focused approach to QA/QC for quantitative MR is required, including broader use of standardized or traceable phantoms, promotion of vendor-neutral QC tools, and early incorporation of QA/QC into emerging AI-assisted workflows. International experience shows that standardized phantoms and automated QC systems significantly improve reproducibility and help detect scanner performance issues at an early stage [20].
4. Finally, a coordinated European effort would benefit from shared resources and collaborative platforms that enable benchmarking, best practice exchange and continuous quality improvement. ESR, as a pan-European scientific society, is well positioned to support these efforts with its partners through guidance documents, educational initiatives and networks that promote harmonization while respecting national specificities [1].

## Conclusions

In conclusion, the survey reveals a diverse and fragmented picture of how MR QA and QC are managed across Europe. The majority of respondents recognize the importance of robust QA/QC programs, citing their role in ensuring patient safety, enhancing diagnostic reliability, reducing errors, and maintaining equipment performance. There is a strong consensus among respondents on the need for harmonized guidelines from organizations like ESR, multidisciplinary collaboration and easily accessible training. Through collaboration between radiologists, medical physics experts, medical physicists, physicists, radiographers and industry partners, it will be possible to improve interoperability and secure long-term quality and safety in MR practice.

## Data Availability

The data generated during and/or analyzed are available from the institutional author on reasonable request.
